# A computational diffusion model to study antibody transport within reconstructed tumor microenvironments

**DOI:** 10.1186/s12859-020-03854-2

**Published:** 2020-11-17

**Authors:** Ana Luísa Cartaxo, Jaime Almeida, Emilio J. Gualda, Maria Marsal, Pablo Loza-Alvarez, Catarina Brito, Inês A. Isidro

**Affiliations:** 1 iBET, Instituto de Biologia Experimental e Tecnológica, Oeiras, Portugal; 2 Instituto de Tecnologia Química e Biológica António Xavier, Universidade Nova de Lisboa, Oeiras, Portugal; 3 Departamento de Geologia, Faculdade de Ciências, Universidade de Lisboa, Lisboa, Portugal; 4 Instituto Dom Luiz, Faculdade de Ciências, Universidade de Lisboa, Lisboa, Portugal; 5 ICFO, Institut de Ciències Fotòniques, The Barcelona Institute of Science and Technology, Castelldefels, Barcelona Spain

**Keywords:** Antibody diffusion, Tumor microenvironment, 3D in vitro cancer models, Computational modelling, Light sheet fluorescence microscopy

## Abstract

**Background:**

Antibodies revolutionized cancer treatment over the past decades. Despite their successfully application, there are still challenges to overcome to improve efficacy, such as the heterogeneous distribution of antibodies within tumors. Tumor microenvironment features, such as the distribution of tumor and other cell types and the composition of the extracellular matrix may work together to hinder antibodies from reaching the target tumor cells. To understand these interactions, we propose a framework combining in vitro and in silico models. We took advantage of in vitro cancer models previously developed by our group, consisting of tumor cells and fibroblasts co-cultured in 3D within alginate capsules, for reconstruction of tumor microenvironment features.

**Results:**

In this work, an experimental-computational framework of antibody transport within alginate capsules was established, assuming a purely diffusive transport, combined with an exponential saturation effect that mimics the saturation of binding sites on the cell surface. Our tumor microenvironment in vitro models were challenged with a fluorescent antibody and its transport recorded using light sheet fluorescence microscopy. Diffusion and saturation parameters of the computational model were adjusted to reproduce the experimental antibody distribution, with root mean square error under 5%. This computational framework is flexible and can simulate different random distributions of tumor microenvironment elements (fibroblasts, cancer cells and collagen fibers) within the capsule. The random distribution algorithm can be tuned to follow the general patterns observed in the experimental models.

**Conclusions:**

We present a computational and microscopy framework to track and simulate antibody transport within the tumor microenvironment that complements the previously established in vitro models platform. This framework paves the way to the development of a valuable tool to study the influence of different components of the tumor microenvironment on antibody transport.

## Background

The value of antibodies as antitumor therapies has been largely demonstrated over the last two decades [1]. Despite the generalized success, there are still challenges to overcome, such as the largely reported poor tissue penetration and heterogeneous distribution of antibodies within solid tumors [2]. Efficacy of therapeutic antibodies is conditioned by several transport barriers, from systemic administration until reaching the target cells [3]. These barriers ultimately cause a reduction of the therapeutic molecule concentration that reaches the target tumor cells, decreasing its therapeutic effect [3–5]. Specifically within the tumor microenvironment (TME), higher heterogeneity is found when comparing with healthy tissue: tumors present altered vasculature, desmoplastic and inflammatory microenvironment and extracellular matrix (ECM) alterations [6]. Within the ECM, collagen fibers and glycosaminoglycans (GAGs) have been previously described as influencing the transport of therapeutic molecules [7–9]. So, it is crucial to assess antibody transport within this intricate network with high impact on therapy efficiency.

Experimental (i.e. in vitro, in vivo and ex vivo) and computational (in silico) models have been developed to help understand how tumor heterogeneity influences drug distribution within the TME [6, 10, 11]. Those two types of models can and should be combined to develop a comprehensive framework to study and try to answer that question.

Several computational models have been developed over the years to describe and simulate the transport and interactions of drugs within the tumor by considering the main transport mechanisms, such as diffusion and convection, degradation and internalization [10, 12–16]. These models can be used to study the complex interaction between several tumor components and drug pharmacokinetics and distribution. They can represent the tumor with different levels of detail, from a simplistic homogeneous tumor mass to complex heterogeneous non-equally distant cancer cells. However, they do not consider the impact on antibody distribution of specific elements of the TME, such as collagen fibers, that have been reported to have a severe influence in this distribution [7–9].

The assessment and tracking of distribution of drugs in vivo, in tumor tissue or in tumor-like structures or complex cell cultures/tissue mimetics is also technically challenging [13, 17] and typically relies on methods that do not allow real-time tracing of antibody distribution [17–20] due to limitations of microscopy techniques and of the biological sample [17, 21]. Our group has been developing modular 3D cell models of the TME [22, 23]. These in vitro cancer models comprise cancer cells and other cellular components of the TME, such as fibroblasts, encapsulated in alginate matrices. We have shown that long-term culture led to recapitulation of specific TME, leading to phenotypic features of disease progression [22, 23].

In this work, 3D in vitro cancer models were used as an experimental platform to assess antibody distribution within the TME. Light sheet fluorescence microscopy (LSFM) was implemented to perform real-time antibody tracking with high resolution 3D imaging over time, together with low photobleaching of the sample fluorescence [24]. An in silico model of antibody diffusion within the TME, developed specifically as a complement to the 3D in vitro models, was calibrated based on these data. Assuming a purely diffusive antibody transport and considering that binding sites on cell surface become saturated over time, Fick’s law was combined with an exponential saturation equation. The computational model was able to describe the antibody concentration profile observed experimentally with very good agreement. Additionally, we show this platform can be used to generate random spatial distributions of the TME elements (tumor cell spheroids, fibroblasts and ECM fibers) inside the capsule, following a tunable stochastic approach.

## Results

### Experimental observation of antibody diffusion through 3D capsule

**Fig. 1:**
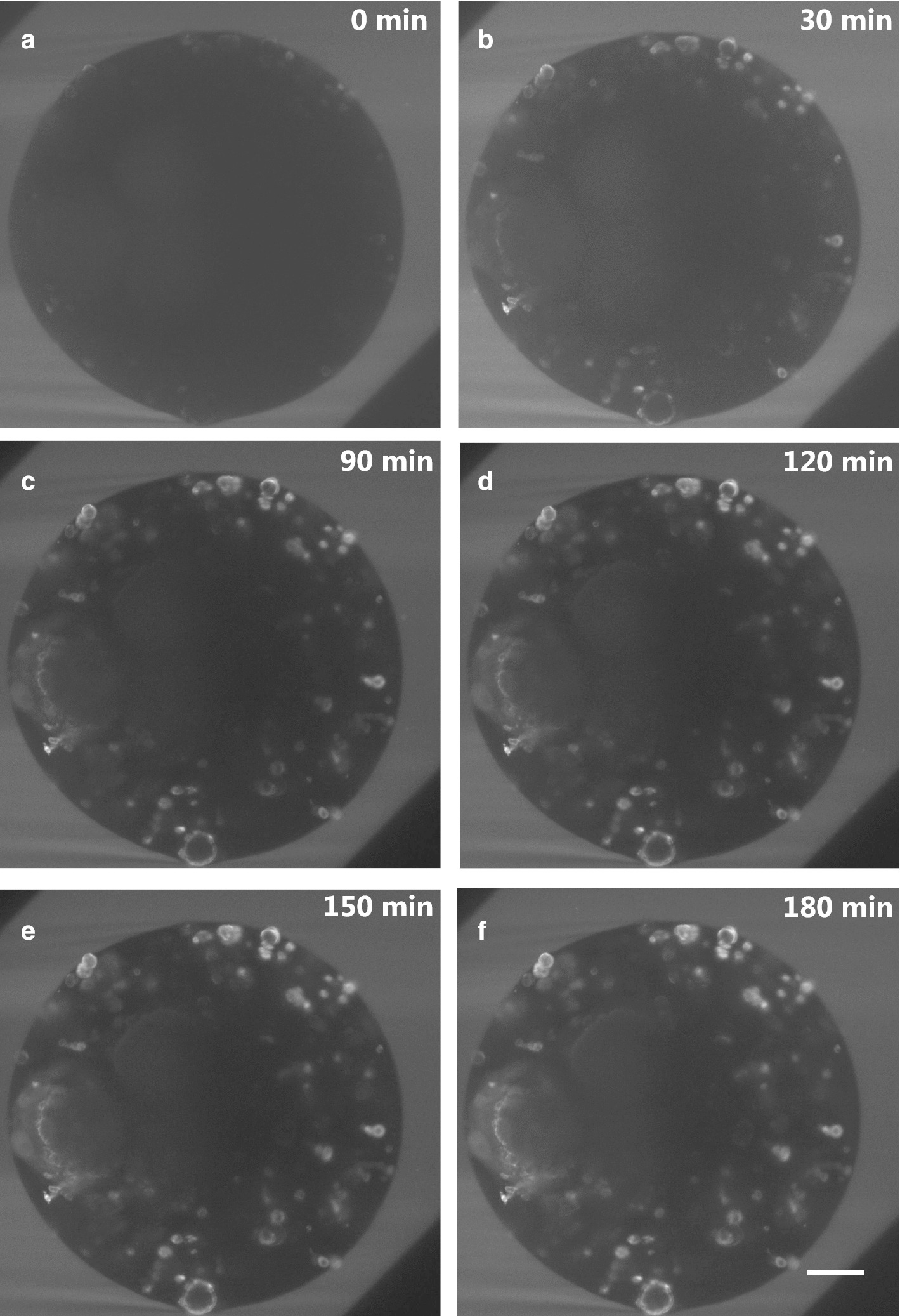
Fluorescence after antibody challenge for a representative capsule section. **a** 0 min; **b** 30 min; **c** 90 min; **d** 120 min; **e** 150 min; **f** 180 min after the antibody challenge; scale bar: 100 µm

Antibody transport within the alginate capsule was tracked using a custom-made LSFM setup. A fluorescent anti-CD44 antibody was used to challenge the encapsulated co-culture of tumor cells and fibroblasts. Over time, fluorescent signal was increasingly detected in cells within the capsules, following a radial trend from the periphery to the inside of the capsule (Additional file 2: Movie 1). The central plane of the 3D capsule acquisition was selected to allow visualization and model calibration (Fig. 1, Additional file 3: Movie 2).

**Fig. 2:**
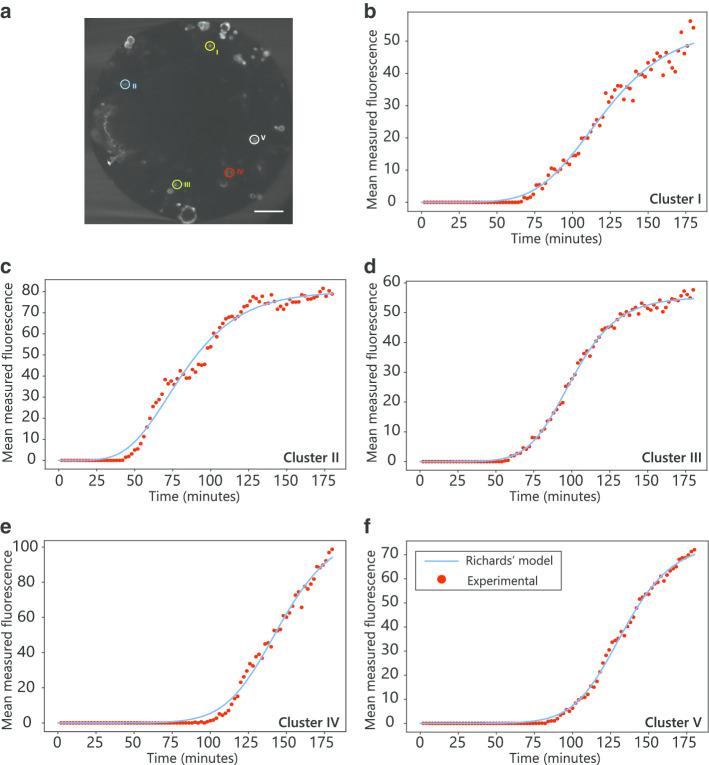
Fluorescence profiles for selected cell clusters and fitted curves. **a** Definition of selected cell clusters (scale bar: 100 µm); **b**–**f** Experimental fluorescence profiles from LSFM data, averaged over the whole cell cluster section (red dots), and fitted curves (blue lines) for the selected cell clusters I through V, respectively. Curve parameters for Eqs. (1–2) are shown in the Table 1

Image processing was used to trace antibody fluorescent signal profiles in defined regions of interest (ROIs), corresponding to cell spheroids or clusters of few cells (Fig. 2a). Despite signal fluctuations, that can be a consequence of sample drift during the LSFM acquisition, cell movement or changes in cell morphology (Additional file 3: Movie 2), these profiles follow a general S-shaped curve (Fig. 2b–f). For cell cluster II, close to the capsule periphery, we observed a fast increase in fluorescence intensity, which stabilized early into a plateau (Fig. 2c). For cell cluster V, further away from the periphery, we observed a delay in the increase in fluorescence intensity and the plateau was reached at least 30 min later (Fig. 2f).

The delay time had a positive correlation with the cell cluster distance to the capsule periphery, although with a high variability (Fig. 2b-f, Figure S1A). It is reasonable to expect the presence of heterogeneous physical and biological barriers in the antibody diffusion path which can influence the delay time. For example, cluster III was farther away from the periphery than cluster I, but both show a similar delay (Figure S1A).

The experimental fluorescence profiles were fitted with mathematical models for S-shaped curves, often used to describe population growth [25]. All selected clusters had the best fit, with R^2^ > 0.98, with the Richards model, given by

1 $$P \left( t \right) = \frac{M}{{\left[ {1 + \alpha \exp \left( { - M \beta t} \right)} \right]^{\gamma } }}$$

$$\alpha = \left[ {\left( {\frac{M}{{P_{0} }}} \right)^{{\frac{1}{\gamma }}} - 1 } \right] \exp \left( {M \beta t_{0} } \right)$$

in which *P* is the mean fluorescence and *α*, *β*, *γ* and *M* are constants [25]. In this study, *M* represents maximum mean fluorescence (upper asymptote), while *β* (intrinsic growth) and *γ* (asymmetry in relation to the inflection point) describe the binding dynamics of the antibody to the antigen on the surface of cells [25, 26].

The fitted smoothed curves describe well the overall fluorescence intensity profiles (Fig. 2, Table 1). Nonetheless, for some of the cell clusters, when the fluorescence intensity becomes detectable, the adjusted curve showed a slight bias towards a shorter delay than was seen in the experimental data (Fig. 2c, e). Consistently with the previous observations, the curve parameters do not follow a clear trend depending on the cluster distance to the capsule periphery or the cluster size.

**Table 1:** Properties for selected cell clusters and parameters for the adjusted fluorescence profiles

Cell cluster	Distance to capsule periphery (μm)	Area (μm^2^)	Fitted parameters			
			M	β	γ	R^2^
I	83.0	232	54.1	6.42 × 10^–4^	4.40	0.987
II	88.1	270	80.2	4.88 × 10^–4^	15.1	0.988
III	115.0	211	55.2	10.1 × 10^–4^	2.44	0.998
IV	124.7	265	109	4.40 × 10^–4^	1.87	0.998
V	127.3	439	75.0	7.09 × 10^–4^	1.78	0.993

### Computational model emulates antibody transport within the capsule

A digitization approach was used to obtain a capsule section equivalent to the one used in the experimental study (Figure S2A-C). The initial diffusivities (Figure S2D) were set taking into account the range of values for this parameter reported on the literature [27–30] and the experimental results over time (Fig. 1). By changing *D*_*medium*_ the “radially moving front” of the antibody distribution can be controlled. Based on experimental observations *D*_*medium*_ was fixed at 0.15 μm^2^/s and *D*_*cell*_ was subsequently fixed at 0.0015 μm^2^/s.

**Fig. 3:**
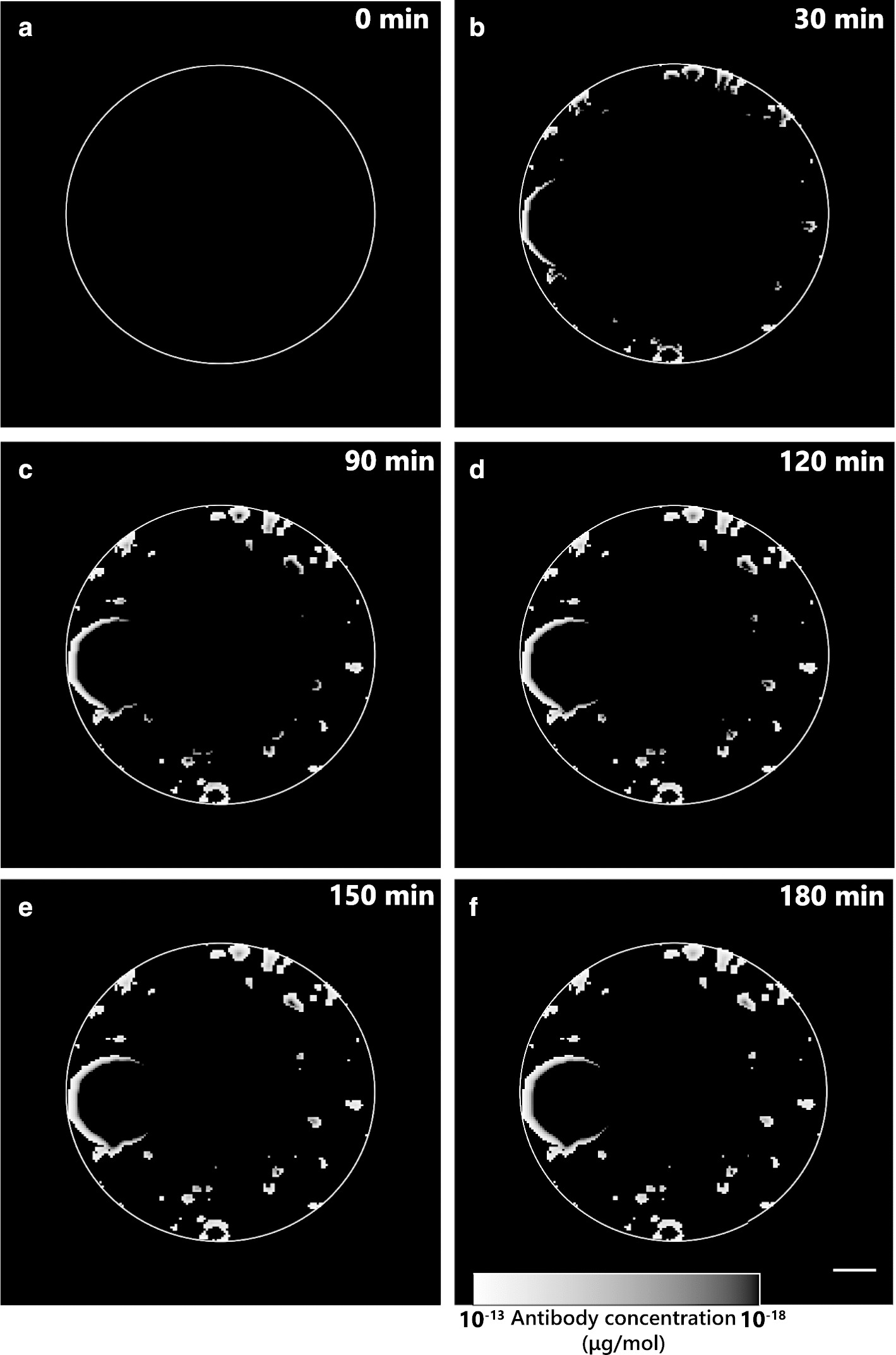
Simulated antibody concentration profile throughout the digitized capsule, over time. Computational images for selected timepoints using saturation parameters *a* = 1, *n* = 1 and *p* = 1: **a** 0 min; **b** 30 min; **c** 90 min; **d** 120 min; **e** 150 min; **f** 180 min; white circumference represents the capsule periphery; scale bar: 100 µm

The computational model combining Fick’s law with exponential saturation, as described in the “Methods” section in Eqs. (3) and (4), has antibody distribution profiles over time which are very similar to the ones obtained experimentally (Fig. 3, Additional file 4: Movie 3). The model diffusivity coefficients decrease over time on the exposed surface of the cell spheroids, as imposed by the saturation equation (Figure S3, Additional file 5: Movie 4).

**Fig. 4:**
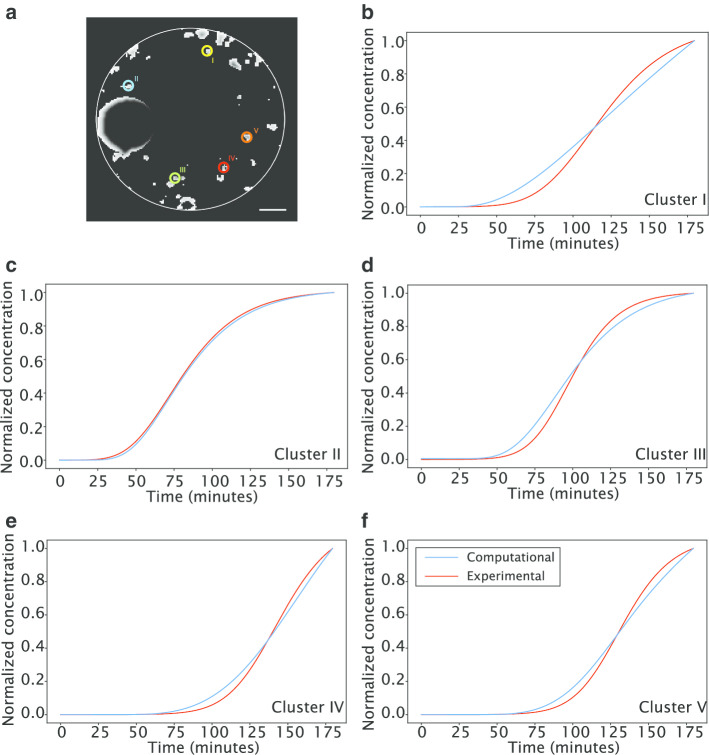
Computational antibody concentration profiles after fitting of the saturation parameters a, n and p to selected cell clusters. **a** Identification of selected cell clusters within the digitized capsule corresponding to the experimental clusters (scale bar: 100 µm); **b**–**f** Experimental mean fluorescence profiles smoothed from experimental LSFM data (red line) and fitted computational curves (blue line) for selected cell clusters I through V, respectively

Antibody concentration profiles in the cell clusters were adjusted to account for binding and saturation of the antigens. To fit the computational model, the parameters of the saturation equation, Eq. (4), were adjusted by minimizing the root mean square error (RMSE) between the normalized experimental and computational profiles, for the 5 selected cell clusters (Fig. 4a). The proposed model was able to represent the experimental profiles with an RMSE up to 5% (Fig. 4b–f and Table 2). The best parameters varied between cell clusters, even for clusters with similar distance to the capsule periphery such as clusters I and II and clusters IV and V.

**Table 2:** Fitted saturation parameters for the computational model and RMSE

Cell cluster	a	n	p	RMSE
I	1.01	1.73	1.33	0.05
II	1.00	1.00	1.00	0.01
III	1.13	0.53	1.34	0.04
IV	0.43	1.91	1.40	0.03
V	0.79	1.57	1.42	0.03

Even though a 5% RMSE was considered low, the systematic deviation observed consistently in the same direction across cell clusters (lower log phase slope for the computational model) suggests the influence of biological or biophysical mechanisms not considered in the model. Consistently, representing signal delay as function of cluster distance to capsule periphery showed that the computational delay is smaller than the experimental by approximately 10 min (Figure S1A). Comparing the slope against cluster area showed that, with the exception of cluster II, there is an approximately constant deviation between the computational and experimental models (Figure S1B).

The computational model was also tested without the saturation equation, Eq. (4). The sigmoidal profile observed experimentally cannot be replicated with any combination of the adjusted parameters, in this case *D*_*cell*_ and *D*_*medium*_ (Figure S4 and Table S1), meaning that a purely diffusive model is unable to explain the behavior observed in the experimental runs.

### Implementation of a modular framework: tuned random distribution of TME elements inside the capsule

The framework used to create the capsules allows us to simulate several distinct but equivalent capsules, i.e. with the same number of cancer cell spheroids and fibroblasts, but with different distribution. It is based on a tunable stochastic algorithm, as detailed in the “Methods” section, which mimics the observed experimental distributions. Along with the cancer cells and fibroblasts, we also included a representation for collagen fibers. Fibril forming collagens are highly abundant in solid tumors, becoming increasing crosslinked and linearized along cancer progression and leading to increased compaction and stiffening of the ECM [31]. Therefore, in the two-dimensional representation of the capsule, we defined collagen fibers as a linear barrier. Some examples of the versatility of this framework are shown in Figure S5.

**Fig. 5:**
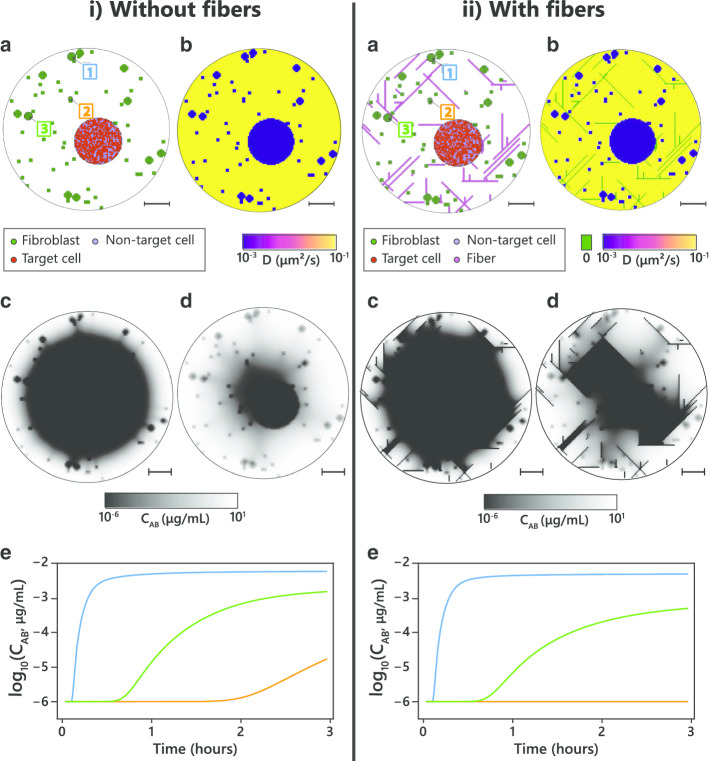
Example of a tuned stochastic computational capsule with and without fibers. Simulation with D_medium_ = 0.15 μm^2^/s, D_cell_ = 0.0015 μm^2^/s, a = 1, n = 1, and p = 1, for two scenarious: **i** without fibers and **ii** with fibers. **a** Graphical representation of one random tuned capsule, with the indication of the selected clusters; **b** Initial diffusivity coefficients throughout the capsule; **c**–**d** Antibody concentration for two different time points (30 and 180 min, respectively); **e** Antibody concentration profile for the three cell clusters identified in **a** (blue—cluster 1, orange—cluster 2, green—cluster 3); scale bar: 100 µm

This framework was tested in silico by creating two identical capsules, one with and one without collagen fibers (Fig. 5a, scenarios i and ii respectively). The diffusivity coefficient for the collagen fiber was set as zero (total barrier), as it has been reported that this ECM component hinders antibody diffusion [7–9], while the remaining TME elements maintained the previously described diffusivity parameters (Fig. 5b). Antibody concentration distributions were compared for both capsules for the second and last time frames (Fig. 5c, d). For the capsule without fibers, the antibody diffuses radially and homogeneously to the interior of the capsule (Fig. 5c, d, scenario i). When fibers are added, antibody distribution throughout the capsule is highly heterogeneous as fibers perpendicularly aligned to the diffusion direction retain the antibody (Fig. 5c, d, scenario ii). This difference is also clear in the antibody concentration profiles for the three selected clusters in each scenario (Fig. 5e). Cluster 1 is near the periphery and the antibody diffusion profile is very similar for both settings. Cluster 2 is in the internal portion of the capsule and is surrounded by fibers that impede diffusion, which in turn virtually nullifies the antibody concentration. Finally, cluster 3 displays an intermediate situation, the antibody concentration profile in the presence of fibers follows a similar trend than in the absence of fibers but with lower maximum.

This theoretical simulation shows the impact of heterogeneous antibody retention within the ECM in antibody transport to the cell. Additional experimental data from capsules containing co-cultures with distinct collagen ECM contents is necessary to validate the representation of collagen and other ECM components.

## Discussion

In the present work, we developed a computational model that reproduces in silico antibody transport within a 3D in vitro cancer model. The in silico model was trained with live tracking data of a fluorescent antibody, generated by LSFM. This microscopic technique allowed us to assess which cells within the alginate capsules, were binding to the antibody and to what extent. LSFM overcomes the limitations of classical fluorescent microscopy techniques, as it provides a good optical sectioning for volumetric rendering, being less aggressive to fluorophores and sample, reducing photo-bleaching and phototoxicity [24].

Experimental results show that the time delay until a detectable fluorescence intensity was attained for each cell cluster has a weak linear correlation with cell cluster distance to the capsule periphery. This hints at the capsule interior being an anisotropic medium with some degree of heterogeneity. Molecules secreted by the cells, such as collagens and glycosaminoglycans, cannot be detected under the microscope and cause antibody retention [7–9], being likely responsible by some of the observed gaps between the computational and experimental models. Additionally, it may be associated with heterogeneous antibody presentation, in particular because there are two cell types being analyzed together.

We assumed a purely diffusive mechanism of antibody transport, i.e. antibody transport is controlled exclusively by concentration gradients. Convection was considered negligible since no significant flow of culture medium was imposed on the experimental setup. The lack of significant flow implies a near zero Péclet number [32] and, therefore, corresponds to a diffusion-driven transport. However, convection could be incorporated in the model for different experimental conditions. The antibody diffusion coefficient in the capsule (*D*_*medium*_) is lower than reference values found in the literature, which range from 0.6 μm^2^/s [28] to 13 μm^2^/s [29]. Both works used tumors models implanted or grown in mice to estimate the diffusivity of a fluorescent antibody, the first across the blood vessel walls and in the interstitium, the second within the vessels around the tumor region. The lower diffusivity should be the result of combined resistance by the alginate network and the ECM deposition within the capsule [22, 23].

Kinetic equations for antibody binding described in the literature [33, 34] require a priori knowledge of kinetic parameters, such as binding and dissociation constants. Because these values are not always available, we opted for a simpler approach, where a generic exponential equation was used to describe the saturation of binding sites as the antibody concentration increases and less antigens become available. This generic approach means the computational model can also be applied directly to study drugs other than antibodies. Our simulated antibody distribution profiles showed that binding site saturation can be correctly represented using this approach. The saturation parameters *a*, *n* and *p* control the shape of the sigmoidal curve (Figure S6). Parameters *n* and *p* change the sigmoidal curve by controlling its slope. So, *n* and *p* can be biologically correlated with ease of antibody binding to the cell cluster. As *n* increases, a longer time is needed to observe any reduction in the diffusivity coefficient. As such, *n* can be correlated with the initial contact of the antibody to the cell cluster, when the antibody concentration is very small. Conversely, *p* controls the stages closer to saturation, when a much higher antibody concentration is present on the cell clusters. Parameter *a* controls mainly the time required to reach the plateau. Thus, *a* can be correlated with the amount of available binding sites on the cell cluster or with binding velocity. *Ergo*, cell clusters with smaller *a* value require a higher antibody concentration to bind to all the available binding sites.

Computational model parameters were optimized to fit each selected cell cluster. This means that, for each fitted model, all clusters were given the same *a*, *n* and *p* parameters as the selected cluster. Following this procedure, we observed that different parameters fit different clusters. It was not possible to find a combination of *a*, *n* and *p* that provided a good fit (RMSE < 5%) for all the cell clusters simultaneously. A modest but systematic deviation was observed in the simulated fluorescence profiles for all cell clusters, which suggests mechanisms that are not being considered are interfering with the antibody transport. Parameters for the saturation equation should be consistent across cell clusters, which suggests ECM components should be assessed experimentally together with antibody distribution so their impact in antibody transport can be included in the computational model. Expanding the 2D in silico model into a 3D representation of the full capsule is also likely to reduce inconsistencies among cell clusters by considering diffusive transport through space and objects in adjacent planes.

The combined experimental and computational framework here presented can be used as basis for future work, exploring experimental models composed of distinct tumor cell lines and additional tumor microenvironment cellular components, resulting in different ECM compositions. This will allow to study antibody transport throughout specific TME features, and give a relevant measure its heterogeneity and defining factors.

## Conclusions

We describe a computational model that reproduces antibody transport within an in vitro tumor microenvironment model, containing different cellular components. Moreover, we showed that the combination of 3D in vitro cell models and light sheet fluorescence microscopy enables the experimental assessment of therapeutic antibody distribution within the tumor microenvironment. Drug molecules with different properties (different sizes and charges), ranging from small molecules such as chemotherapeutic drugs, to larger molecules such as antibodies, can be studied using the same approach.

Ultimately, the combined experimental and computational framework can be employed not only to decipher how different elements within the TME can influence drug transport, but also, once that influence is understood, to work as a predictive tool. This would help reducing experimental burden and costs by performing a computational screening of specific conditions prior to experimental testing.

## Methods

### Experimental setup, data collection and processing

#### Cell lines and 2D cell culture

NCI-H157 (#CRL-5802; from now on referred as H157) Non-Small Cell Lung Carcinoma (NSCLC) cell line was obtained from American Type Culture Collection (ATCC). Human Dermal Fibroblasts (hDFs) isolated from human skin were obtained from Innoprot. Cell were routinely screened for mycoplasma contamination by PCR (Eurofins Genomics Europe Applied Genomics GmbH, Germany). Cells were cultured under 2D static conditions, maintained at 37 ºC in an incubator with humidified atmosphere containing 5% CO_2_ and 21% of O_2_.

Tumor cells were sub-cultured twice a week with a seeding density of 1 × 10^4^ cell/cm^2^ and maintained in Tumor Cell Culture Medium, composed of Dulbecco's Modified Eagle Medium (DMEM) supplemented with 1 mM sodium pyruvate (Life Technologies), 12 mM HEPES (Life Technologies) and 0.1 mM non-essential amino acids (Life Technologies). hDFs were split once a week, at a seeding density of 0.5 × 10^4^ cell/cm^2^ and cultured in Iscove's Modified Dulbecco's Medium (IMDM, Life Technologies) supplemented with 10% (v/v) fetal bovine serum (Gibco) and 100 U/mL penicillin–streptomycin (Gibco).

#### Cell microencapsulation and stirred suspension culture

H157 cell spheroids were generated in all-baffled spinner-flasks with a straight blade paddle impeller (Corning Life Sciences), according to the aggregation protocol previously established in-house [35]. Spheroids were collected 3 days after spinner inoculation for the establishment of co-cultures, as described previously [23]. Briefly, 2 × 10^4^ spheroids were mixed with a single cell suspension of hDFs and the mixture was dispersed in 1.1% (w/v) of Ultrapure Ca^2+^ MVG alginate (UP MVG NovaMatrix, Pronova Biomedical), dissolved in 0.9% (w/v) NaCl solution [22, 23]. Microencapsulation was performed using an electrostatic bead generator (Nisco Encapsulator) with an air flow rate of 10 mL/h, at 5.3 V with air pressure of 1 bar, to generate capsules of approximately 700 μm; alginate droplets were cross-linked in a 20 mM BaCl_2_ bath. Encapsulated co-cultures were maintained for 2 weeks under agitation (shake flasks, 80 rpm), with medium exchange twice a week (half of the volume replaced by fresh Tumor Cell Culture Medium), to allow time for the build up of TME features [23].

#### Light sheet fluorescence microscopy setup

All the images were acquired with a custom-made LSFM system, an improved version of the SPIM-fluid system [36]. The illumination path consists in three CW lasers with excitation wavelengths of 488, 561 and 637 nm (Cobolt; MLD 50 mW, DPL 100 mW and MLD 150 mW, respectively). Laser beams are expanded using a telescope system, composed of two achromatic doublets [Thorlabs, AC254-050-A-ML (f = 50 mm) and AC254-200-A-ML (f = 200 mm)], creating a flat top Gaussian beam profile. The light sheet is created by a pair of galvanometric mirrors (Thorlabs, GVSM002), which pivoting planes are properly conjugated with the back focal aperture of the objective lens (Nikon, 4x PlanFluor NA 0.13). Double side illumination is achieved by duplicating these elements and adding a 50/50 beamsplitter cube (Thorlabs, CCM1-BS013). A relay lens set, with two achromatic lenses [Thorlabs, AC254-075-A-ML (f = 75 mm)] is used as a bridge, so optical planes are properly conjugated in the right arm. In the detection path, a water dipping objective (Nikon 10x 0.3) is used to collect the generated fluorescence from the top of the incubation chamber, as in an up-right microscope configuration. An achromatic doublet with focal distance of 200 mm (AC254-200-A-ML) is used to form the image onto the sCMOS camera chip (Hamamatsu Orca Flash4.0). Different emissions are selected using a motorized filter wheel (Thorlabs, FW102C), equipped with three filters (Chroma and Semrock: 520/15 (GFP), 590/50, 638LP (Cell tracker deep red)). The scanning of the sample is performed by translation of the whole physiological chamber with a motor (PI M-501.1DG) through a fixed horizontal light sheet plane (Figure S7). The Flexi-SPIM microscope features a custom developed software based on LabVIEW (National Instruments). This software allows the user to access settings of the various devices on a single graphical user interface. An Arduino UNO board, connected via USB to our workstation, is integrated in the LabVIEW software providing control of the shutters, bright-field illumination and sample rotation. The custom-made LSFM allows for different possibilities for the sample mounting. In order to provide flexible, fast and easy-to-use sample loading capabilities, an imaging chamber was designed and 3D printed using fluorinated ethylene propylene (FEP). FEP presents a refractive index similar to water (1.33) and is CO_2_ permeable. So, it allows for live imaging on specimens using water-dipping objectives. Prior to imaging acquisition, samples were loaded into FEP tubes and transported towards the detection objective field of view using a programmable syringe pump (Tecan, Cavro Centris). Once here, the two motors can rotate the sample, in order to choose the view of interest. This mounting system offers the possibility to easily insert, aspirate and discard the specimens without the need of agarose, enabling the possibility for high-throughput studies with relatively big samples (up to 1 mm diameter).

#### Antibody challenge, image acquisition and processing

The fluorescent antibody (anti-CD44 Monoclonal Antibody (IM7), PE, eBioscience) was diluted in Tumor Cell Culture Medium, to a final concentration of 13 µg/mL, close to the range of therapeutic antibody concentration found in patient serum [37, 38].

Individual capsules were harvested from culture and loaded into the microscope tube in order to obtain a control image of the intrinsic autofluorescence and to assess general capsule features (in the bright-field). The capsule was then removed from the FEP tube, immersed in the antibody solution and reloaded in the microscope. Fluorescence was acquired in the 561 nm laser line. A 3D stack of the capsule containing 235 planes separated by 3 µm, covering 705 µm, was acquired every 2 min. Acquisition was performed over a total of 3 h. Images were acquired with a pixel size of 0.65 µm.

The fluorescence intensity profiles for selected cell clusters were obtained from the central plane of the capsule. Each cluster was marked using a ROI defined by a circular domain. The fluorescence intensity over time was integrated and converted to a profile. All image processing was performed using Image J (Rasband, W.S., ImageJ, U. S. National Institutes of Health, Bethesda, Maryland, USA, https://imagej.nih.gov/ij/, 1997–2018), version 1.52i. Further details on image processing are described in the Supplementary Methods (Additional file 1).

To smooth the experimental data noise, the five cluster profiles were fitted to growth curve models [25] and the model type showing the best fit (highest R^2^) was selected. For comparison between the profiles of the selected clusters, the delay time and the slope of log phase of the curve were calculated. The first of these parameters can be seen as a measurement of the time required for the antibody to reach that cell cluster in sufficient concentration to be detected. It was calculated as the time interval from the beginning of the experiment until the mean fluorescence value became larger than 5% of the total achieved fluorescence for that selected cluster. The slope of the log phase was obtained by calculating the slope of a linear fit, adjusted for this phase alone. For consistency, we assumed log phase of the curve to correspond to the portion of the model that takes place when the measured value (i.e. fluorescence and antibody concentration) corresponds to 15–85% of its maximum observed value.

### Antibody diffusion model within the alginate capsule

Antibody transport within the alginate capsule was assumed to be purely diffusional. In fact, assuming the capsule is immersed in a static fluid, no relevant convective transport is expected to occur [39]. This diffusive behavior is modelled by Fick’s second law, which in bidimensional space is defined by

3 $$\frac{\partial C}{{\partial t}} = D\left( {\frac{{\partial^{2} C}}{{\partial x^{2} }} + \frac{{\partial^{2} C}}{{\partial y^{2} }}} \right)$$

in which *D* is the diffusion coefficient (*D*_*medium*_ for the medium and *D*_*cell*_ for the cells), *C* is the concentration of the antibody, *t* is the time, and *x* and *y* are the Cartesian coordinates.

Cells detain a limited number of antigens where antibodies can bind. So, as the antibody binds, the number of available binding sites on the cell surface is reduced until reaching saturation, in which all or the vast majority of the binding sites are bound to an antibody molecule. This biological effect can be translated in terms of variation of diffusivity of the antibody in the cells and was assumed to follow an exponential saturation curve described by

4 $$D_{cell}^{t + \Delta t} = D_{cell}^{t} \times \exp \left( { - a \times \frac{{C_{norm}^{n} }}{{\left( {1 - C_{norm} } \right)^{p} }}} \right)$$

in which *a*, *n* and *p* are adjustable parameters, *D*_*cell*_ is the diffusivity coefficient in the cells and *C*_*norm*_ is a normalized concentration. The latter takes into account the fact that the concentration at which the cells get saturated is much smaller than the antibody concentration in the medium and is given by

5 $$C_{norm} = \frac{{C_{i,j}^{t} }}{{C_{inj} \times 0.01}}$$

in which *C*_*inj*_ is the antibody concentration of injection and *C*^*t*^_*i,j*_ is the concentration of antibody in a cell localized in the position *i, j*.

The model proposed here works under the following assumptions: (i) no significant antibody degradation occurs; (ii) the initial antibody concentration inside the capsule is zero; (iii) cell growth and death are not relevant; (iv) cell movement is neglected; (v) ECM formation, degradation or re-structure is negligible within the time interval of the experiment; (vi) initial *D*_*cell*_ was set as being 100 smaller than initial *D*_*medium*_ for all cells (single cells, cancer cell spheroids and fibroblasts) and (vi) *D*_*medium*_ is constant over time and through the extracellular space inside the capsule.

Additionally, we also consider that antibody diffusivity within the spheroid varied with the spheroid radius according with

6 $$D_{cell}^{d} = D_{cell}^{\max } \times \left( {\frac{d}{{r_{sph} }}} \right)$$

in which *D*_*cell*_^*d*^ is the diffusivity coefficient of a cell in the spheroid whose distance to the cancer spheroid center is *d*, *D*_*cell*_^*max*^ is the diffusivity coefficient of the cells located in the outer layer of the spheroid and *r*_*sph*_ is the spheroid radius.

### Computational model fitting and simulation

#### Definition of the initial setup: capsule domain

**Fig. 6:**
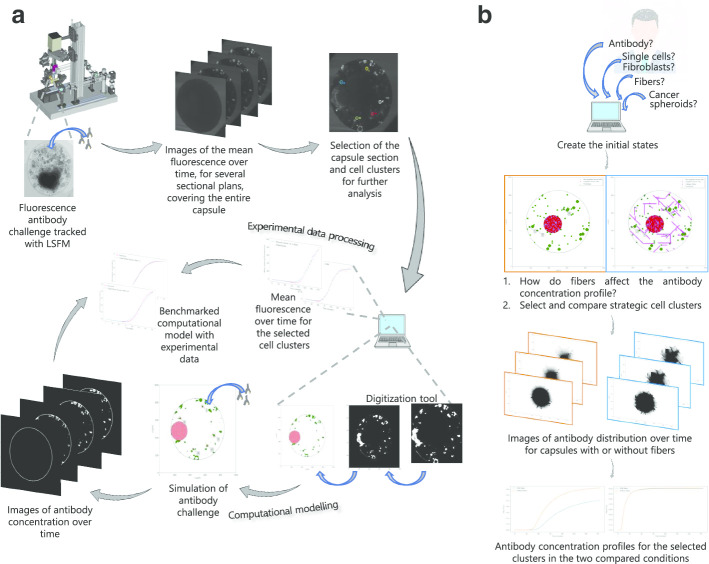
Experimental and computational workflow. **a** Methodology applied in the digitized capsule approach (LSFM: light sheet fluorescence microscopy). **b** Methodology applied in the tunable stochastic approach on an example

The computational model and all simulations were implemented and run in Python (version 3.7). To numerically solve Fick’s second law in Eq. (3), the finite differences method was applied. This method to solve differential equations requires a discretized domain. So, a two-dimensional square grid (mesh) with 200 × 200 equally spaced nodes and a 1000 µm side was created. Method convergence was evaluated as described in Supplementary Methods section and Figure S8 (Additional file 1). Each element (node) was assigned a range of intrinsic attributes (coordinates: x and y; type: cancer cell, fibroblast or ECM; diffusivity; and concentration). This grid is further split between two major domains: a central circular domain, representing the alginate capsule (centered in the mesh and with a radius of 350 µm) and its surrounding medium, representing the culture medium outside the alginate capsule. For the distribution of the TME elements within the digital capsule, two alternative approaches were used (Fig. 6). In one approach, the distribution was obtained by digitization of an experimental capsule slice. The alternative approach entailed the random distribution of TME elements by the application of a tuned algorithm based on theoretical assumptions.

#### Capsule digitization from experimental capsule images

Digitization of the capsule allowed to reproduce the experimental capsule in silico, with cancer cell spheroids and fibroblasts localized in the same positions. Based on the last frame of the experimental antibody diffusion video for a specific capsule slice (Additional file 3: Movie 2), Python Imaging Library (PIL) was used to convert the figure into a binary input (Fig. 6a). The cancer cell spheroid was added manually, according with the contrast phase images acquired experimentally.

#### Tunable random distribution of TME elements

A stochastic framework, in which the TME elements are randomly distributed inside the capsule was also implemented. This process is tunable by the user who can set the total number and preferential distribution of each TME element to include in the capsule (Fig. 6b). To exemplify this modular nature, two capsules were created, both with the same cancer cell and fibroblast distribution but only one included collagen fibers. Cancer cells were allocated on a single circular cell cluster, which was randomly placed within the capsule. Fibroblasts were randomly distributed, with a preference towards the outer ring (comprising 20% of the capsule radius), both as single cells and as small clusters. They were defined with a radius of 11.5 µm [40] and 17.5 µm (from experimental LSFM images) for single cells and clusters, respectively. The cell number and distribution were based on theoretical assumptions from the observation of several capsule slices and previous data [23]. Collagen fibers were randomly distributed, within the unallocated capsule space and were assumed to have 30 μm length [41], 4 possible orientations (0°, 45°, 90°, 135°) and zero diffusivity. The overall process of model development is schematized in the workflow in Figure S9 and Fig. 6b.

#### Benchmark of the computational model with experimental data

Experimental fluorescence profiles and computational antibody concentration profiles were normalized so as to vary between 0 and 1 and thus allow their comparison (Fig. 6a). The benchmarking of the computational model by the experimental data was performed by implementing the Broyden-Fletcher-Goldfarb-Shanno (BFGS) optimization algorithm to find the saturation parameters *a, n* and *p* of Eq. (4) which minimize the root mean square error (RMSE) for a set of selected cell clusters. The RMSE between the experimental and computational normalized profiles is given by

7 $${\text{RMSE = }}\sqrt {\mathop \sum \limits_{i = 1}^{n} \frac{{\left( {\widehat{{y_{i} }} - y_{i} } \right)^{2} }}{n}}$$

in which *n* is the number of points in which computational and experimental values are compared, *ŷ*_*i*_ is the predicted value from the computational model and *y*_*i*_ is the observed values in the experiments.

All cells were assumed to be target cells of the antibody, since CD44 is detected in both hDFs [42] and H157 cells [43].

#### Boundary conditions

The capsule external domain was defined as having a fixed antibody concentration of 13 μg/mL, as in the experimental setup. We assume that, for the modelled timeframe, the depletion of the medium is not significant since the volume of antibody solution is two orders of magnitude higher than the capsule volume.

## Supplementary information

**Additional file 1:** Supplementary information (Supplementary Figures S1–S9, Supplementary Table S1 and Supplementary Methods).

**Additional file 2: Movie 1.** Video of the LSFM maximum intensity projection for all acquired frames, corresponding to 3 h.

**Additional file 3: Movie 2.** Video of the LSFM selected capsule central plane, corresponding to 3 h.

**Additional file 4: Movie 3.** Video of the simulated antibody concentration over the 3 h time interval.

**Additional file 5: Movie 4.** Video of the evolution of simulated diffusion coefficients over time throughout the capsule.

## Data Availability

The dataset supporting the conclusions of this article is included within the article and its additional files.

## Abbreviations

BFGS: Broyden–Fletcher–Goldfarb–Shanno
ECM: Extracellular matrix
FEP: Fluorinated ethylene propylene
GAG: Glycosaminoglycan
hDFs: Human Dermal Fibroblasts
LSFM: Light sheet fluorescence microscopy
NSCLC: Non-Small Cell Lung Carcinoma
PIL: Python Imaging Library
RMSE: Root mean square error
TME: Tumor microenvironment
