# Supplementary material for: A computational diffusion model to study antibody transport within reconstructed tumor microenvironments
Source: BMC Bioinformatics. 2020 Nov 17;21:529. doi: 10.1186/s12859-020-03854-2 (PMC7672975; doi:10.1186/s12859-020-03854-2)
Supplement: Supplementary file 1 — Additional file 1: Supplementary information (Supplementary Figures S1–S9, Supplementary Table S1 and Supplementary Methods). [file 12859_2020_3854_MOESM1_ESM.pdf]

A computational diffusion model to study antibody transport within reconstructed tumor microenvironments

AL Cartaxo, J Almeida, EJ Gualda, M Marsal, P Loza-Alvarez, C Brito, IA Isidro

Supplementary information

Supplementary tables

Table S1: Fitted D_{cell} and D_{medium} by application of the BFGS algorithm, assuming a purely diffusive mechanism without cell saturation.

Cell cluster	D_{medium} ($\mu m^2/s$)	D_{cell} ($\mu m^2/s$)	RMSE
**I**	0.164	0.126	0.06
**II**	0.659	0.110	0.09
**III**	0.712	0.156	0.11
**IV**	0.151	0.103	0.04
**V**	0.210	0.166	0.06

Supplementary figures

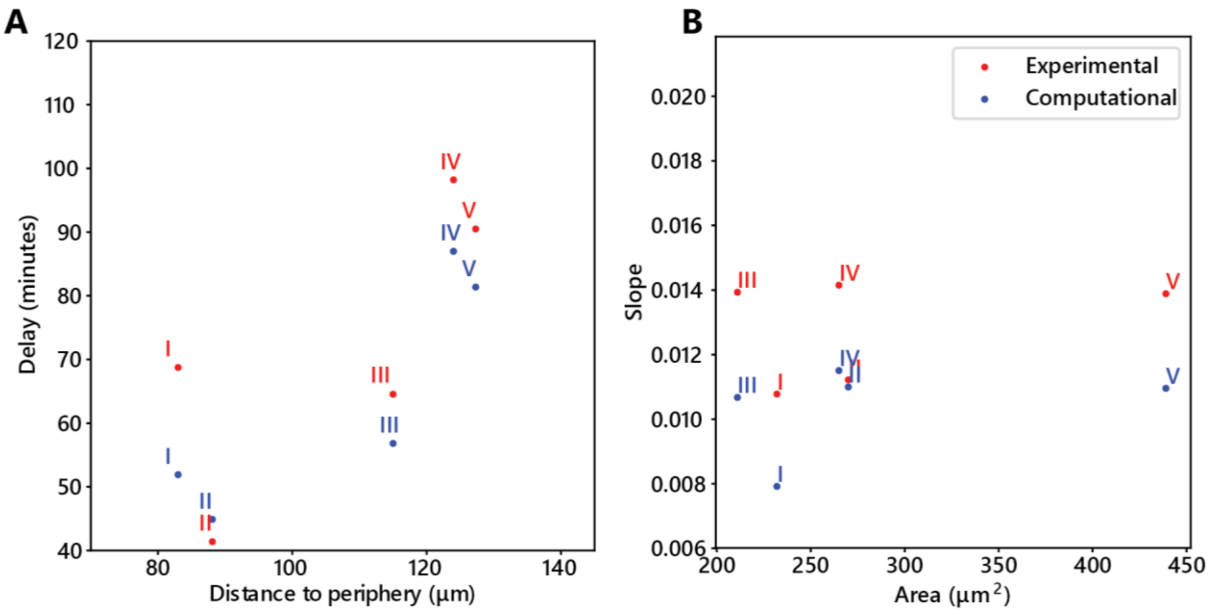

Figure S1: Comparison of profile features (delay time and slope) with cell cluster features (distance to capsule periphery and section area) obtained experimentally and for the computational model. **A)** Experimental delay vs distance to periphery. **B)** Slope of log phase of the curve vs area of the cell cluster.

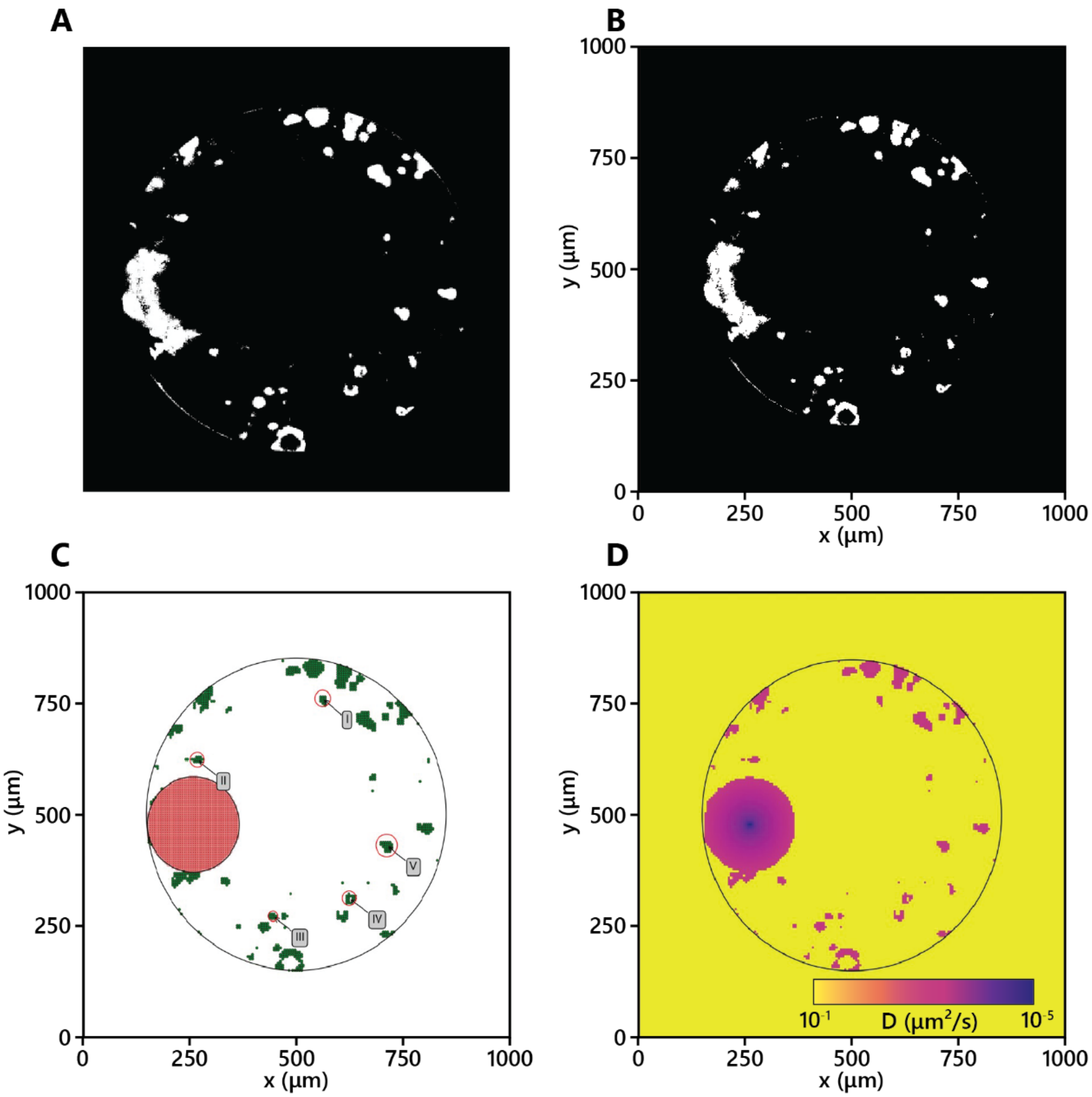

Figure S2: Definition of the initial setup by application of Python Imaging Library (PIL) tool. **A)** Read-out from Fiji of the last frame for the selected capsule section; **B)** digitized capsule obtained by application of PIL tool to A); **C)** component distribution and aggregate placement in accordance with B) and identification of the selected cell clusters equivalent to the experimental capsule section (Figure 2A); **D)** Diffusivity coefficient distribution within the capsule in the computational model for the digitized capsule.

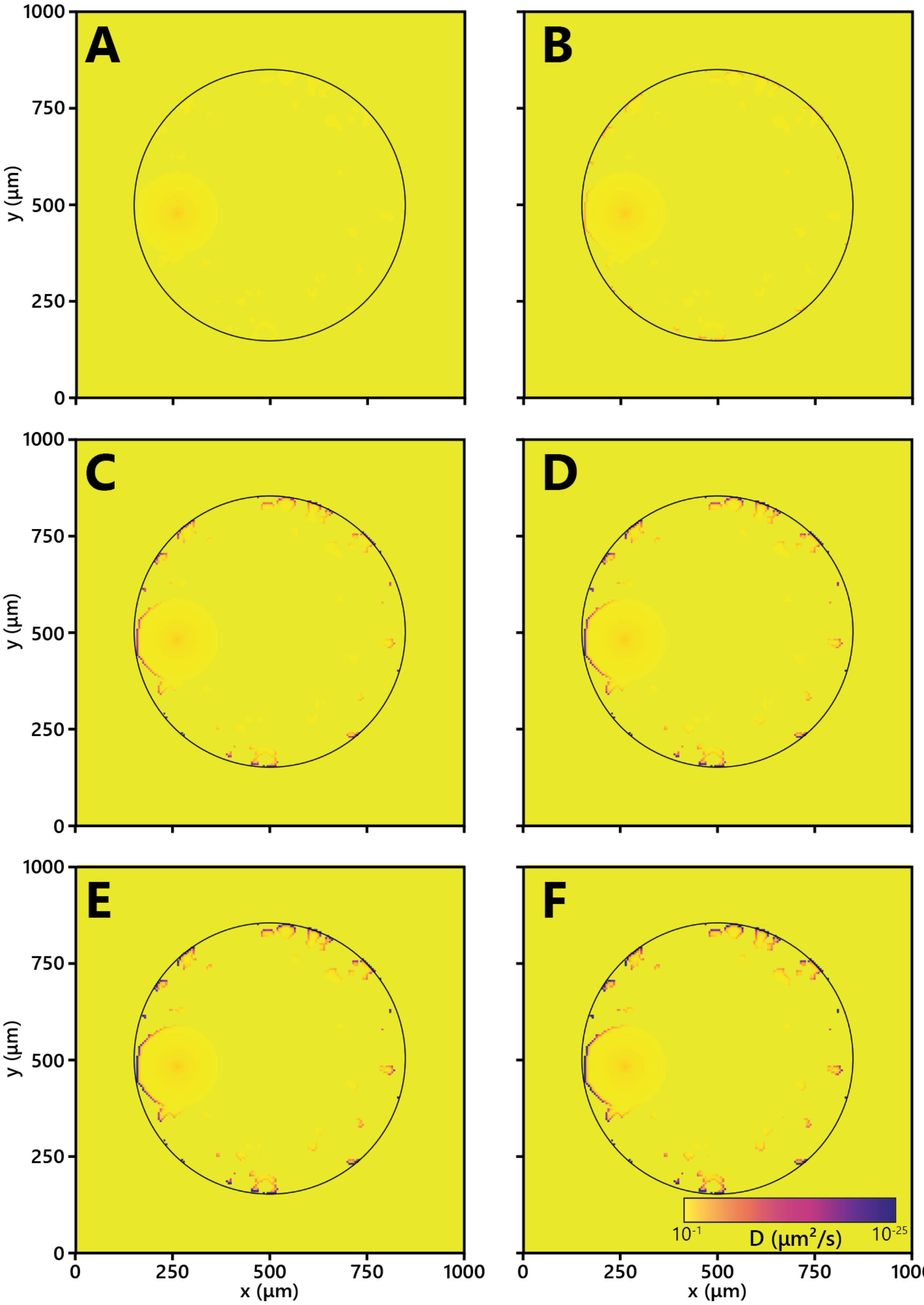

Figure S3: Diffusivity coefficient on the computational model over time, for the digitized capsule. Computational model was run for a=1, n=1, p=1: **A)** 0 min; **B)** 30 min; **C)** 90 min; **D)** 120 min; **E)** 150 min; **F)** 180 min.

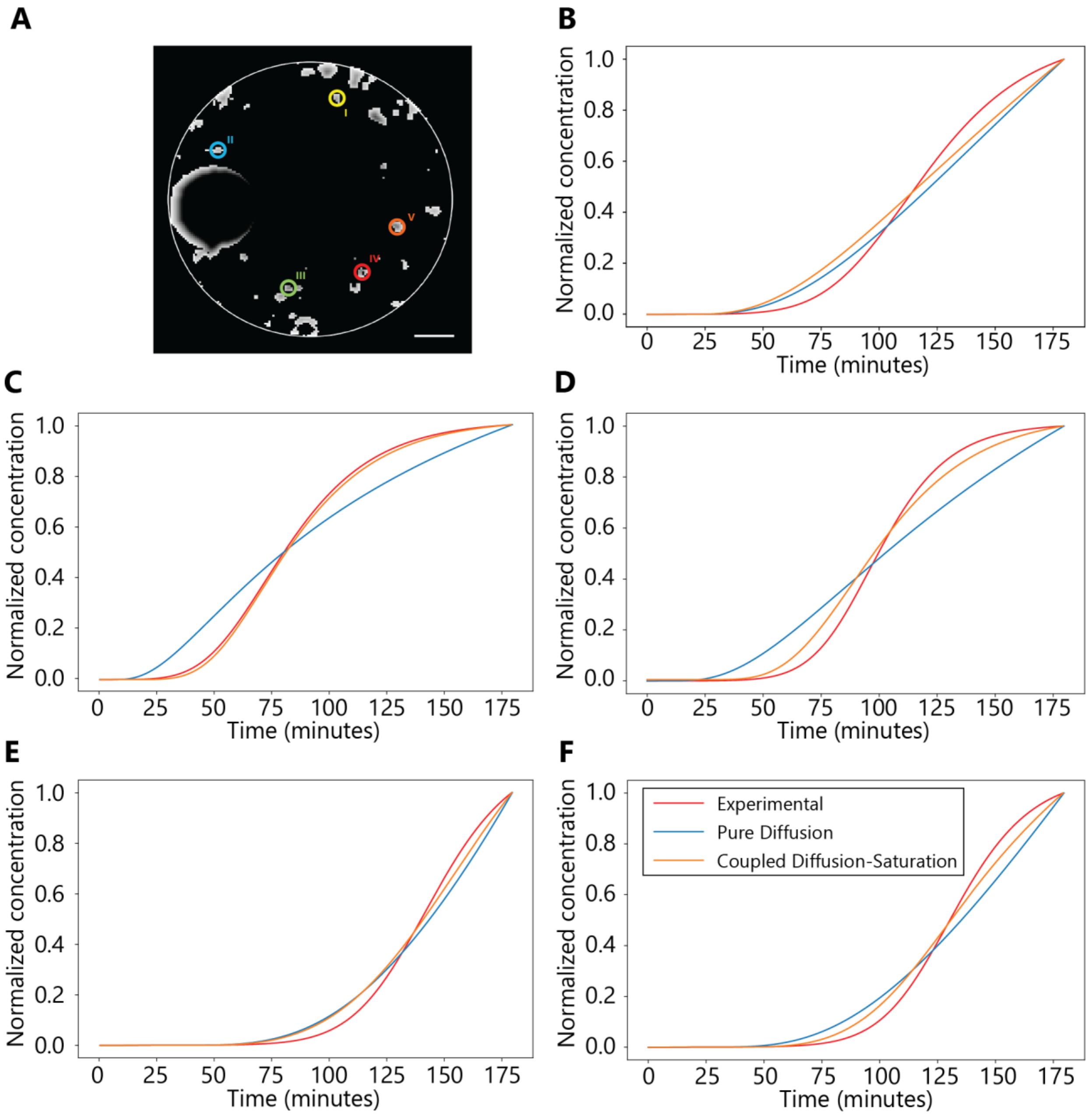

Figure S4: Comparison of best fittings obtained considering only Fick's law (blue curve) or Fick's law combined with exponential saturation (orange curve) to the experimental data (red curve) and corresponding RMSE *scale bar: 100 µm.*

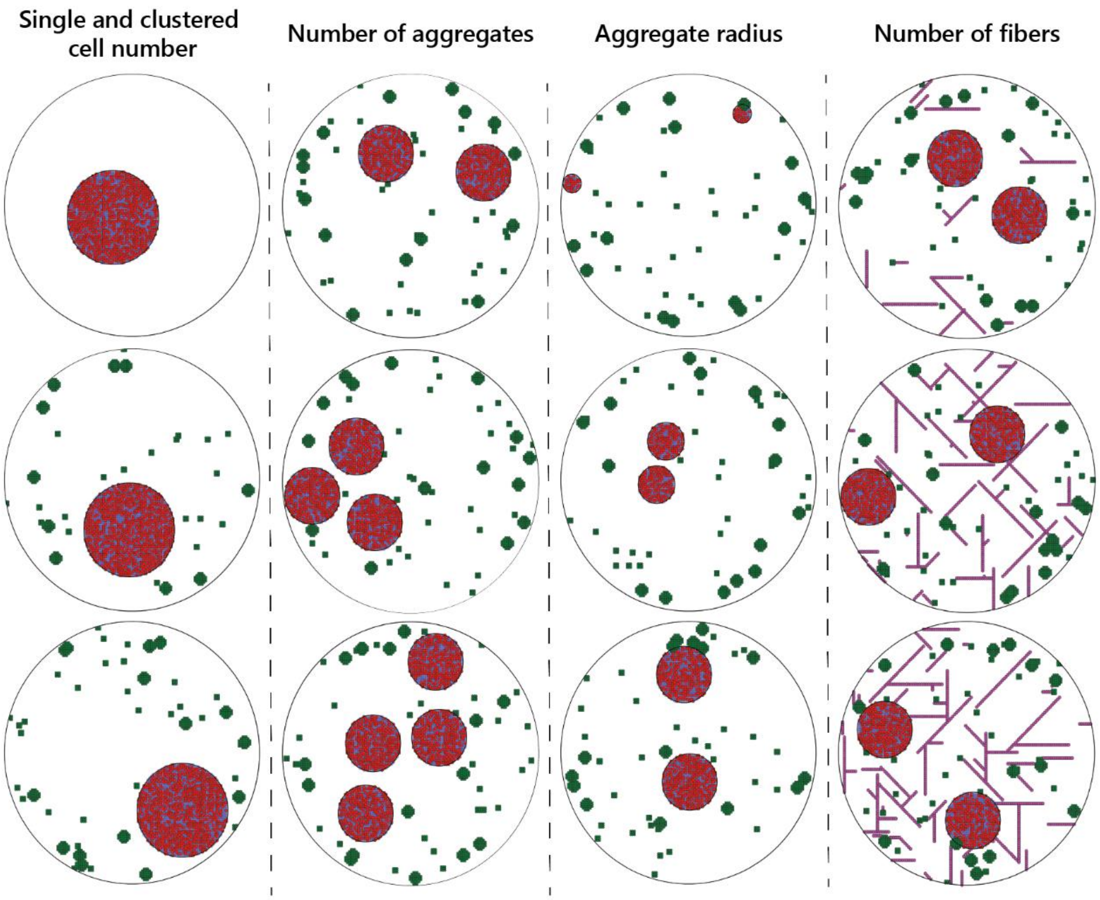

Figure S5: Tunable stochastic computational model examples. A modular tuned stochastic computational framework was developed and its versatility allows the user to set a defined amount of cancer cells aggregates and fibroblasts as single cells or clusters and also collagen fibers. First column shows models with varying amount of single and small clustered cells. Second column shows capsules with different number of aggregates. The third column shows variation in cancer aggregate radius. The fourth column shows the inclusion of several quantities of collagen fibers.

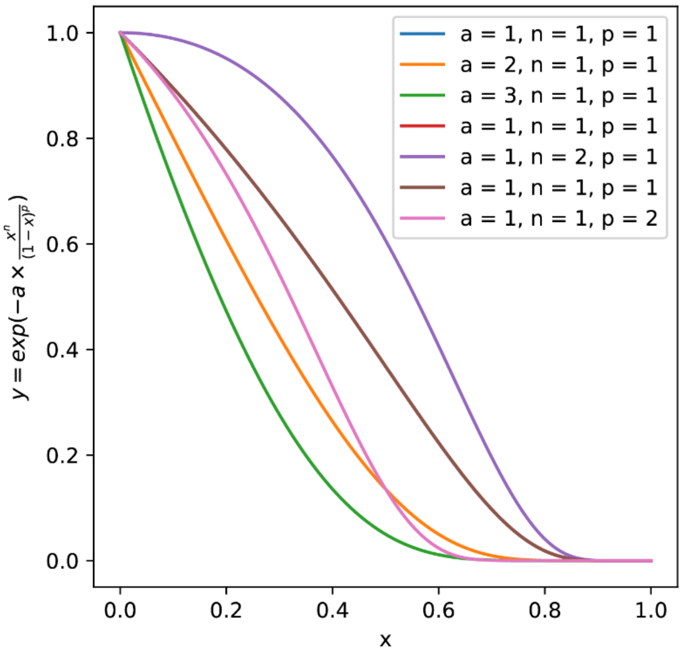

Figure S6: Exponential saturation equation for several different inputs.

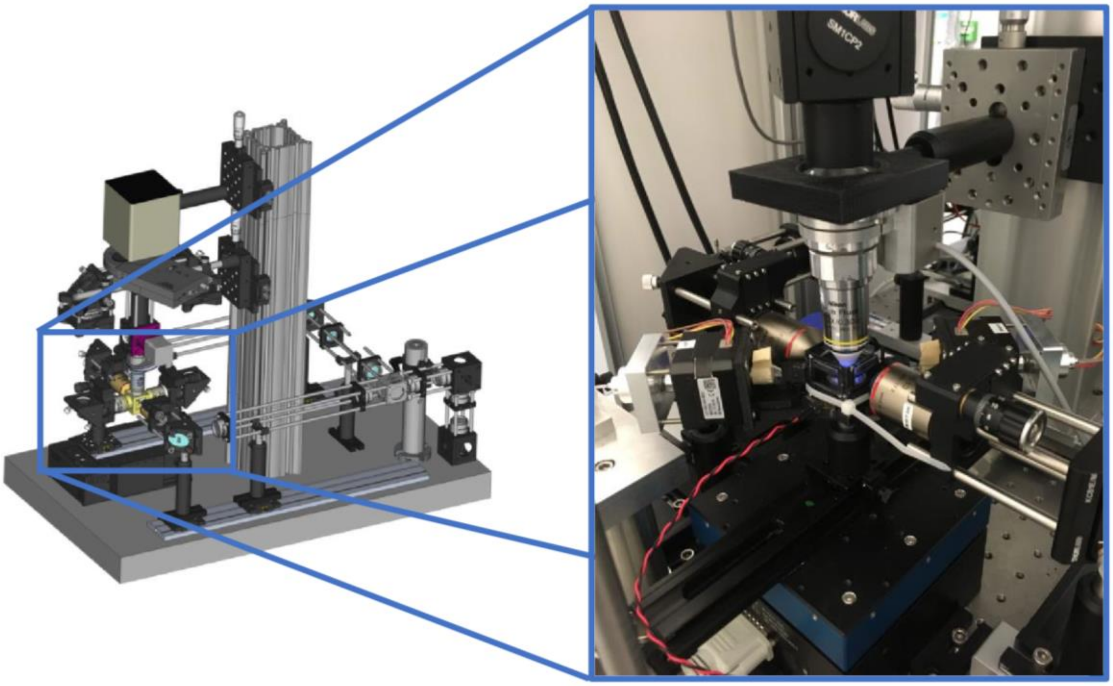

Figure S7: Schematic representation of the LSFM acquisition portion with a photography showing a zoom on the FEP chamber. Lasers illuminate the sample (loaded on a TEP tube) from two directions. The acquisition camera is located 90º in relation with the lasers plan.

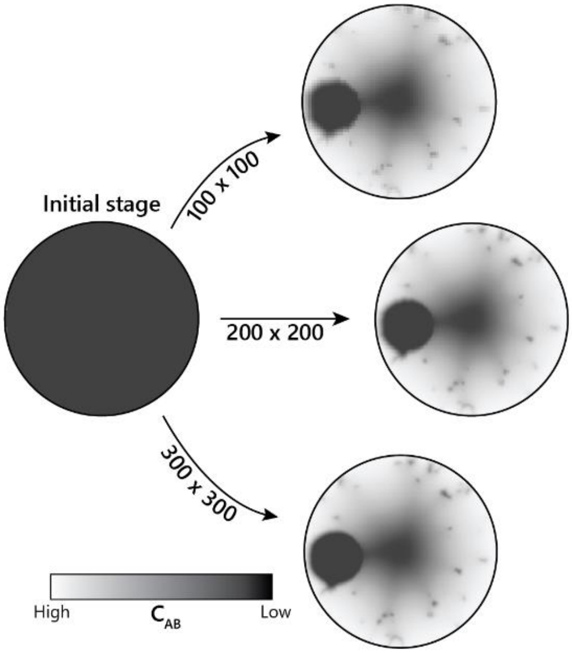

Figure S8: Mesh convergence study: 100x100, 200x200 and 300x300. Initial and final time-points for the model run with the specific mesh size.

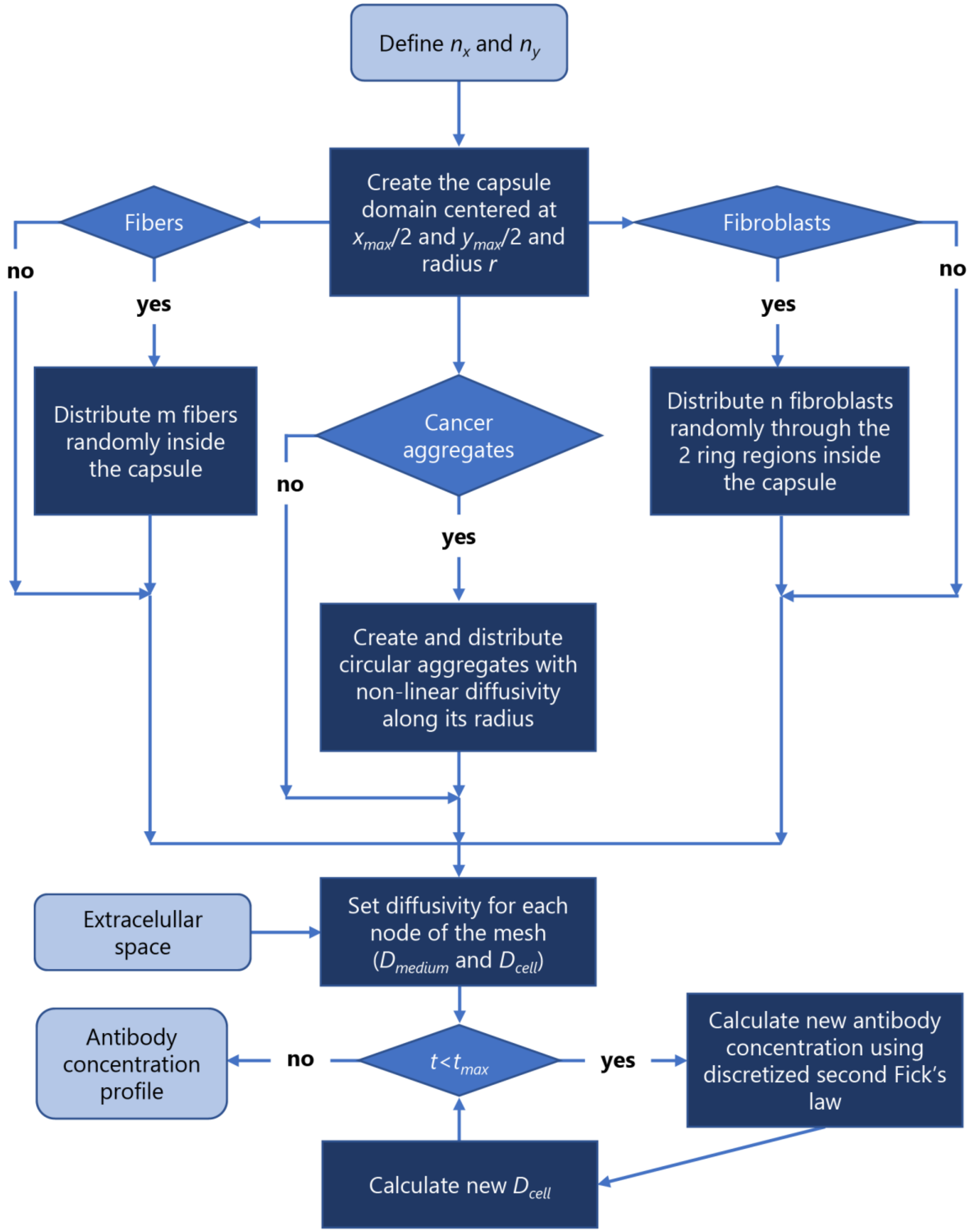

Figure S9: A flow diagram of the tunable stochastic computational framework. The model starts by defining the mesh and creation of the domain. Then, verifies if fibroblasts, cancer aggregates and fibers are to be included in the model. Then, the remaining free space in the mesh is defined as the extracellular space and the diffusivity for all the parts of the system is set. Then, it calculates the concentration of the antibody in each node of the mesh by using the discretised second Fick's law. With these new values, it calculates the new cell diffusivity coefficients. This process runs iteratively until reaching the maximum time, in which it closes the cycle and creates the antibody concentration profiles.

Supplementary Methods

S1. Image processing procedure

All image processing was performed using Fiji, ImageJ software (Rasband, W.S., ImageJ, U. S. National Institutes of Health, Bethesda, Maryland, USA, https://imagej.nih.gov/ij/, 1997-2018). Data collected from LSFM experiments were sets of sequential images of the fluorescence of several sections of each alginate capsule over time. For further analysis, only one capsule section corresponding to middle plan was selected. First, we selected regions of interest (ROI) corresponding to the areas where cluster of cells were identified. The image was converted to *8-bit*, a threshold mask was applied and the image was transformed into binary. The diffusion profiles (mean grey values over time) were obtained using the Z-axis profiler plugin. Five representative cell clusters of one capsule were selected for this analysis.

S2. Method convergence with varying mesh size

A method convergence study was performed choose the mesh size. Different mesh sizes were tested to assess if the obtained antibody concentration profiles were not significantly affected by increasing the mesh step. The step process was the following: 1) create a mesh using the fewest, reasonable number of elements and analyze the antibody concentration profile inside the capsule; 2) recreate the mesh with a denser element distribution, and compare the new obtained antibody concentration profiles to those obtained with the previous mesh. If the final antibody distribution throughout the capsule is equivalent in the tested conditions, then the mesh size selected for the study was thin enough to give accurate results. In this study, we compared meshes with size (number of nodes in x and y) of 100x100, 200x200 and 300x300, maintaining the 1000 µm side (Figure S8).
